# Atypical Cardiac Compression Technique on a Seriously Skeletal Deformity Child

**DOI:** 10.5152/eurasianjmed.2023.0226

**Published:** 2023-06-01

**Authors:** Halil Keskin, İbrahim Halil Başaslan, Hafsa Elif Çobanoglu, Sümeyye Al

**Affiliations:** 1 Division of Pediatric Intensive Care Unit, Ataturk University, Faculty of Medicine, Erzurum, Turkey; 2 Department of Pediatrics, Ataturk University, Faculty of Medicine, Erzurum, Turkey; 3 Department of Anesthesiology and Reanimation, Ataturk University, Faculty of Medicine, Erzurum, Turkey

To the Editor

The cardiac compression techniques were explained in the past by Pediatric Basic and Advanced Life Support 2020 American Heart Association Guidelines.^1^ In addition, Jung et al^2^ described the “Knocking-fingers” technique that was not in this guide. After reading this article with interest, we also experienced that this technique is effective in infant cardiac arrests. We could not find in the literature the chest compression technique we had to apply to a patient in our tertiary pediatric intensive care unit. This previously diagnosed Spinal Muscular Atrophy type 2 and a glial tumor 13-year-old boy developed cardiac arrest. It was impossible to apply effective cardiac compression with known techniques for him because of his serious skeletal deformities (Figure 1). For this reason, we applied to the patient a cardiac compression technique we developed spontaneously at that time (Figure 2). The patient was gotten in a semi-sitting position (30-45˚). The rescuer fixed the patient’s right shoulder with her right hand. She made a fist with her left hand. She applied the periodic pressures perpendicularly to the diaphragm under the left costal arch with her fist, just like in the Heimlich maneuver. The resuscitation response was performed with 10-second ultrasound assessments every 2 minutes. The process was successful after the cardiac compression and the third dose of adrenaline administration, which can be done with this technique. This technique may be an alternative to the traditional method for cardiac compressions in patients with severe skeletal deformities.

## Figures and Tables

**Figure 1.**
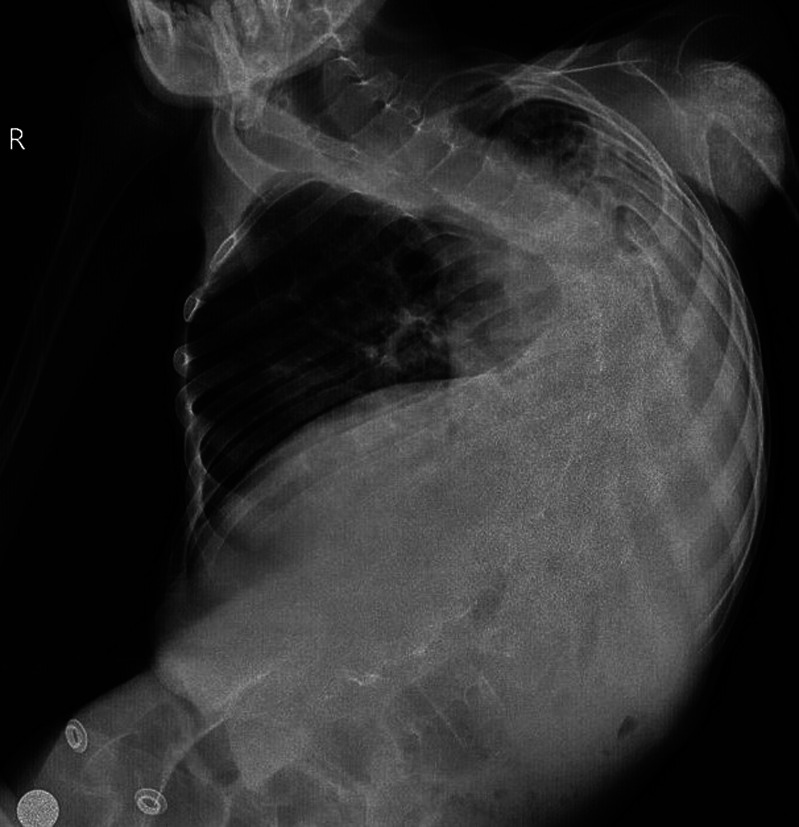
X-ray findings show serious skeletal deformities.

**Figure 2.**
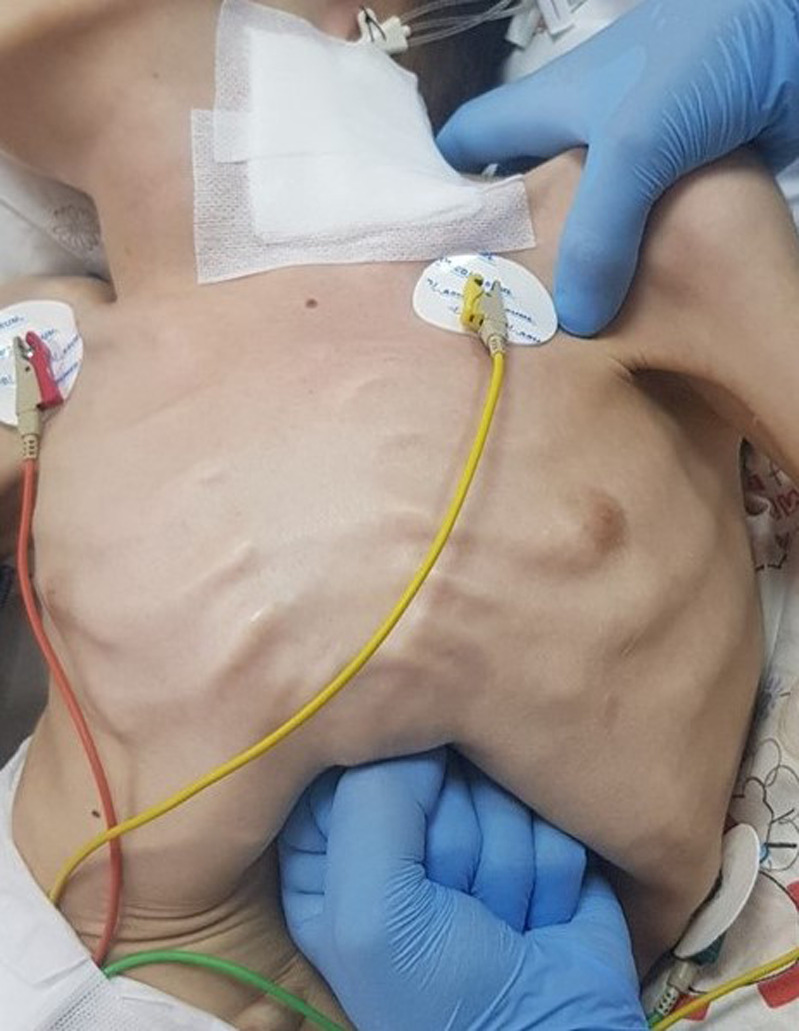
A new cardiac compression technique was applied to the patient by a rescuer.

## References

[1] Topjian AA Raymond TT Atkins D et al. Part 4: Pediatric Basic and advanced life support 2020 American Heart Association guidelines for cardiopulmonary resuscitation and emergency cardiovascular care. Pediatrics. 2021;147(Suppl 1):e2020038505. 10.1542/peds.2020-038505D) 33087552

[2] Jung WJ Hwang SO Kim HI et al. 'Knocking-fingers' chest compression technique in infant cardiac arrest: single-rescuer manikin study. Eur J Emerg Med. 2019;26(4):261 265. 10.1097/MEJ.0000000000000539) 29384754

